# Digestion of Alcoholic Fluids

**Published:** 1847-06

**Authors:** 


*Digestion of Alcoholial Fluids, by Messrs. Saudras and Bouchardat.*—In their previous physiological researches, the authors inquired into the digestion of fat, saccharine and feculent principles, and their share in nutrition; fermented liquids still remained to be studied, and the following are the results of the investigation they have instituted for the purpose of completing the history of what they call "the food of the organs of respiration." During the first part of the process of digestion fermented fluids are not dissolved, but are merely diluted by the saliva, gastric juice, and the other liquids which occupy the cavity of the digestive viscera. As M. Magendie had already shewn, the absorption of the alcoholic fluids is performed by the venous orifices in the stomach, and the lacteal vessels have no share in this action—a circumstance proved by the facts, that after the ingestion of alcohol, not a particle of that substance can be detected in the chyle. The organs of secretion do not eliminate fermented fluids, a small portion of which only, is evaporated from the pulmonary surface. When excessive quantities of spirituous liquids have been admitted into the circulation their first effect is to prevent the arterial blood from acquiring its florid color, and to expose the patient to asphyxia. The oxygen largely introduced into the system during the act of respiration may often convert the alcohol into water and carbonic acid; but in several cases an intermediate product, acetic acid, has been obtained. Fermented fluids, very soon pass entirely out of the body; and their elimination is more speedy when they have previously been in combination with dextrine or glucosis.

